# Comparison of balance, proprioception, and fear of falling in older adults with and without a compensatory dynamic knee valgus

**DOI:** 10.1186/s12891-026-09883-x

**Published:** 2026-04-22

**Authors:** Youssef Mohammad Rahim Al-Saadi, Sajad Roshani

**Affiliations:** 1 Department of Exercise Physiology and Corrective Exercise, Faculty of Sport Science, Urmia University, Urmia, Iran; 2 Department of Exercise Physiology and Corrective Exercise, Faculty of Sport Science, Urmia University, Urmia, Iran

**Keywords:** Dynamic knee valgus, Postural balance, Proprioception, Fear of falling, Fall risk, Older adults rehabilitation

## Abstract

**Background:**

Compensatory movement patterns in older adults may reflect biomechanical abnormalities and sensorimotor system impairments, which can increase the risk of falls. This study aimed to compare static and dynamic balance, knee proprioception, and fear of falling in older men with and without a dynamic knee valgus.

**Methods:**

This causal-comparative study included 60 older men aged 60–70 years from Urmia, Iran. Participants were divided into two groups (*n* = 30 each) with and without a dynamic knee valgus, determined through observational assessment of knee alignment during a squat task. Static balance was evaluated using the Sharpened Romberg test, dynamic balance using the Timed Up and Go (TUG) test, knee proprioception using a photogrammetry-based method, and fear of falling using the Falls Efficacy Scale–International (FES-I). Data were analyzed using independent samples *t*-tests with a significance level set at 0.05.

**Results:**

Participants with a dynamic knee valgus demonstrated significantly poorer performance in all measured variables, including static balance (with eyes open and closed), dynamic balance (TUG), knee proprioception, and fear of falling, compared with those without knee valgus (*p* < 0.001).

**Conclusion:**

A dynamic knee valgus may serve as a clinical may be associated with impaired balance and sensorimotor function in older adults. Early identification of this pattern during screening assessments may be associated with fall-related risk factors and inform the design of targeted sensorimotor corrective interventions.

## Background

In recent decades, declining fertility rates and increased life expectancy have led to substantial changes in the global population structure, resulting in a marked growth of the older adults population [1] Although the threshold for old age is defined as 65 years in international literature, individuals aged over 60 years are considered older adults in Iran [2]. The global population of individuals aged over 60 years has been estimated at more than 700 million and is projected to reach two billion by 2050, a growth rate that far exceeds that of the child population [3]. In line with this global trend, it is anticipated that the older adults population of Iran will increase to approximately 25 million individuals—nearly one quarter of the total population—by 2050 [1] .

Falls are defined as unintentional events that result in an individual coming to rest on the ground [4]. They are the most common adverse events among older adults and are associated with substantial healthcare costs, functional limitations, reduced quality of life, and increased mortality [5, 6]. Additionally, falls can lead to physical disabilities as well as psychological consequences such as fear of falling and reduced independence. Epidemiological evidence indicates that approximately one-third of individuals over 65 years of age experience at least one fall annually, many of whom require medical attention [7, 8].

Physiological and biomechanical changes associated with aging—such as reductions in flexibility, agility, speed, and particularly balance—substantially increase the risk of falling in this population. Among these factors, impaired balance is recognized as one of the primary contributors to falls, disability, and dependency in older adults. Balance is a complex motor skill that reflects the dynamic control of posture required for fall prevention and is essential for performing activities of daily living, playing a critical role in both static and dynamic tasks. In many cases, compensatory movement patterns arising from impaired neuromuscular coordination lead to reductions in muscular strength, movement efficiency, and dynamic balance. These alterations not only limit functional performance but also significantly increase the risk of falling. Furthermore, additional factors such as gait disturbances, neuromuscular abnormalities, use of certain medications, psychological disorders (including depression), cognitive impairments (e.g., memory decline), and visual disorders further exacerbate fall risk [9].

Frontal-plane instability is considered an important factor in dynamic knee valgus, as inadequate control of hip and knee motion in this plane can lead to medial displacement of the knee during functional tasks [10]. Increased postural sway in the medio-lateral direction—particularly at higher movement velocities—is associated with reduced postural stability and increased instability. This phenomenon is closely linked to impairments in proprioception and diminished sensory feedback from the lower extremities [11].

With advancing age, the primary systems involved in balance gradually deteriorate; as a result, the body’s ability to detect shifts in the center of mass and generate appropriate neuromuscular responses to correct postural alignment becomes compromised [12].

One of the key factors influencing postural stability and balance in older adults is alteration in lower extremity alignment, particularly at the knee joint. As the intermediate joint of the lower extremity kinetic chain, the knee plays a critical role in force absorption and weight transfer. Knee valgus, defined as medial deviation of the knee, is a common malalignment that may present structurally or as a compensatory movement pattern resulting from proximal muscle weakness (e.g., hip abductors) or age-related degenerative joint changes. The presence of a dynamic knee valgus can alter gait biomechanics and displace the center of pressure (COP), thereby creating significant challenges in maintaining both static and dynamic balance [13].

In addition, the somatosensory system—particularly proprioception—plays a fundamental role in maintaining joint stability and overall postural control. Mechanoreceptors located within muscles, ligaments, and joint capsules transmit information regarding joint position and movement to the central nervous system, enabling the regulation of appropriate motor responses. Aging is associated with a decline in somatosensory system efficiency and reduced proprioceptive acuity, which may lead to impaired postural control and diminished capacity to respond effectively to balance perturbations. These age-related changes are considered major contributors to increased fall risk and functional instability in older adults [14].

Despite the well-documented roles of balance impairment, proprioceptive deficits, and fear of falling in older adults, the contribution of compensatory lower limb movement patterns— dynamic knee valgus—has received limited attention. From a clinical movement assessment perspective, identifying such compensatory patterns may provide insight into underlying neuromuscular dysfunctions associated with instability and fall risk. Therefore, the present study aimed to compare static and dynamic balance, knee proprioception, and fear of falling in older men with and without a dynamic knee valgus pattern.

## Methodology

### Study design and participants

This study employed a causal-comparative design and was approved by the Ethics Committee of Urmia University (Ethics Code: SSRI.REC-2510-3063). The statistical population consisted of older men aged 60–70 years residing in Urmia, Iran. A total of 60 participants were purposively selected based on the study inclusion criteria and assigned to two groups: individuals with a dynamic knee valgus (*n* = 30) and those without knee valgus (*n* = 30). Written informed consent was obtained from all participants prior to participation.

### Group classification

Participants were classified based on the presence of dynamic knee valgus observed during a standardized squat task. Dynamic knee valgus was operationally defined as medial displacement of the knee relative to the second toe during the descent phase of the squat, assessed in the frontal plane using a two-dimensional observational approach with established reliability and validity [15] and supported by its association with three-dimensional biomechanical contributors [16].

The squat task was performed under standardized conditions. Participants stood with their feet shoulder-width apart and toes pointing slightly outward (~ 10–15°), and performed a squat to approximately 60° of knee flexion. The movement tempo was controlled (2 s descent, 2 s hold, and 2 s ascent) to ensure consistency. Knee flexion depth was visually estimated by the examiner.

Knee alignment was visually assessed by a single trained examiner with prior experience in movement analysis. The assessment was conducted from the frontal plane at a standardized distance of approximately 2 m at hip level. A trial was considered positive if medial knee displacement relative to the second toe was observed. Participants were classified into the valgus group if this pattern was consistently observed in at least two out of three trials, regardless of whether it occurred unilaterally or bilaterally [17].

#### Inclusion criteria were

Male participants aged 60–70 years, no history of falling, no use of tricyclic medications, no history of fracture or dislocation in the lower extremities, and no participation in physiotherapy or regular exercise programs for at least six months prior to the study.

#### Exclusion criteria 

Included unwillingness to continue participation and inability to follow the test instructions.

Static balance was assessed using the Sharpened Romberg test. Participants stood barefoot with one foot placed directly in front of the other, with the dominant foot consistently positioned in front for all participants to ensure standardization. while crossing their arms over the chest. The duration for which the participant was able to maintain this position with eyes open and eyes closed was recorded as the test score. Test reliability has been reported as 0.99 for the eyes-open condition and 0.75 for the eyes-closed condition [18]. Test errors included separation of the arms, displacement of the feet, or excessive body sway; in the eyes-closed condition, opening the eyes was also considered an error.

Dynamic balance was evaluated using the Timed Up and Go (TUG) test. Participants were instructed to rise from an armless chair, walk 3 m toward a marker, turn around it, return to the chair, and sit down again. Timing began at the verbal command “go” and ended when the participant was fully seated. The test was performed three times with one-minute rest intervals, and the shortest time was recorded as the final score. The reliability of the TUG test has been reported as 99% [19]. Based on performance time, scores of < 10 s indicate normal mobility, 10–19 s indicate independent walking, 20–29 s reflect reduced mobility and balance impairment with a need for assistance, and times greater than 30 s indicate severe mobility limitation and a high risk of falling.

Knee joint proprioception was assessed using an active joint position reproduction test under weight-bearing conditions. Reflective markers were placed on anatomical landmarks including the greater trochanter, lateral joint line of the knee, fibular head, and lateral malleolus. An additional marker was placed over the iliotibial band near the popliteal region. Participants wore fitting athletic clothing to ensure accurate placement and visibility of reflective markers.

Participants stood with the dominant foot in contact with the ground while the contralateral limb remained relaxed with the knee flexed. Hands were placed on a support in front of the body to maintain balance.

With eyes closed, participants were instructed to shift their body weight onto the tested limb and move the knee from a neutral position (0°) to a target angle within the range of 40°–60° of knee flexion, holding this position for 5 s. The limb was then returned to the neutral position.

Participants were subsequently asked to actively reproduce the same target angle and maintain it for 3 s. This procedure was repeated three times.

A digital camera was positioned at a distance of 2 m and a height of 70 cm to capture the movement. Images were analyzed using Kinovea software, and joint angles were calculated by connecting the markers. Absolute error values (without direction) were calculated as the difference between the target angle and the reproduced angle across trials [20], which describes the anatomical landmarks and placement of reflective markers. Furthermore, photogrammetry-based assessment of knee joint angles has demonstrated excellent reliability in previous studies (ICC = 0.93–1.00) [21, 22].

Fear of falling was assessed using the 16-item Falls Efficacy Scale–International (FES-I), developed and validated by Yardley et al. This questionnaire evaluates concern about falling during various daily activities such as house cleaning, dressing, shopping, walking outdoors, stair negotiation, and more challenging situations including walking on slippery or uneven surfaces. Each item is scored on a four-point Likert scale ranging from 1 (“not at all concerned”) to 4 (“very concerned”), with higher total scores indicating greater fear of falling. All participants completed the questionnaire, and total scores were calculated by summing item responses [23].

Descriptive and inferential statistical analyses were performed. Data were summarized using measures of central tendency and dispersion (mean ± standard deviation). The Shapiro–Wilk test was applied to assess data normality. After confirming normal distribution, independent samples *t*-tests were used to compare outcome variables between groups. All analyses were conducted using IBM SPSS Statistics version 26 (IBM Corp., Armonk, NY, USA), with the level of significance set at 0.05 [24].

## Results

Descriptive statistics for age, height, weight, and body mass index (BMI) are presented in Table 1.

**Table 1:** Comparison of demographic characteristics between groups

Variable	Valgus (*n* = 30) Mean ± SD	Non-Valgus (*n* = 30) Mean ± SD	t	*p*-value
Age (years)	64.26 ± 2.14	64.10 ± 2.30	0.29	0.773
Height (cm)	169.66 ± 3.69	171.83 ± 4.33	-2.08	0.042
Weight (kg)	74.50 ± 3.80	75.03 ± 4.02	-0.53	0.600
BMI (kg/m²)	25.86 ± 0.61	25.39 ± 0.49	1.31	0.196

Based on the results of the independent *t*-test, none of the variables including age, height, weight, and BMI showed a statistically significant difference between the two groups under comparison (*p* > 0.05).

The results of the independent *t*-test comparing the study variables between older adults with and without a dynamic knee valgus are presented in Table 2.

**Table 2:** Comparison of study variables between older adults with and without a dynamic knee valgus

Variable	t	df	*p*	Group	Mean ± Sd
Static Balance (Eyes Open)	-10.47	46.50	< 0.001	with Valgus Knee	17.20 ± 2.79
				without Valgus Knee	27.86 ± 4.82
Static Balance (Eyes Closed)	-10.93	58	< 0.001	with Valgus Knee	6.26 ± 2.09
				without Valgus Knee	12.56 ± 2.35
Dynamic Balance	11.06	43.89	< 0.001	with Valgus Knee	18.40 ± 3.31
				without Valgus Knee	10.83 ± 1.74
Proprioception	6.90	58	< 0.001	with Valgus Knee	6.0 ± 1.28
				without Valgus Knee	3.60 ± 1.40
Fear of Falling	5.63	35.16	< 0.001	with Valgus Knee	25.53 ± 6.63
				without Valgus Knee	18.36 ± 2.17

These findings indicate significant differences between older adults with and without a dynamic knee valgus across all examined variables (*p* < 0.001)

## Discussion

The findings of the present study demonstrated that older adults with a compensatory knee movement pattern— dynamic knee valgus—exhibited significantly poorer performance in static and dynamic balance, knee proprioceptive accuracy, and greater fear of falling compared with their healthy counterparts. These findings are consistent with previous clinical evidence reported by Sadeghi et al. [25], Rahimi et al. [26], and Hasanvand et al. [27], and further highlight the interrelated contributions of lower limb alignment abnormalities, muscular imbalance, proprioceptive deficits, and psychological factors to increased fall risk in older adults.

From a kinetic chain perspective, dynamic knee valgus may be associated with neuromuscular factors involving adjacent joints. For example, previous studies have suggested that hip and ankle muscle function may play a role in lower limb alignment during functional tasks. However, these variables were not directly assessed in the present study and should therefore be interpreted as potential contributing factors rather than confirmed mechanisms [28]. The presence of these factors may be associated with altered postural control and increased fear of falling.

Additionally, knee valgus alters the orientation of the ground reaction force (GRF) vector relative to the knee joint center, leading to lateral displacement of the force vector and generation of abnormal rotational moments around the sagittal axis [29]. Compensation for these abnormal moments requires increased neuromuscular activation; however, this compensatory capacity is often limited in older adults due to weakness of pelvic stabilizing muscles, particularly the gluteus medius [30, 31].

Supporting this mechanism, Sorkhe et al. demonstrated that strengthening hip abductors improves GRF components and reduces dynamic knee valgus during functional tasks [30], underscoring the regulatory role of pelvic musculature in lower limb stability [32]. Moreover, knee valgus frequently coexists with excessive foot pronation [33], further compromising stability within the closed kinetic chain. This interaction has been indirectly supported by Beyranvand et al. [34], who reported a direct association between ankle proprioception and postural control, emphasizing that dysfunction at any joint within the kinetic chain can negatively affect overall balance [25, 28].

At the sensory–neural level, the present findings indicate reduced proprioceptive accuracy in older adults with knee valgus. This observation aligns with the theoretical frameworks proposed by Riemann and Lephart [35] and Ghotbi and Hassanpour [36]. In valgus alignment, medial knee structures such as the medial collateral ligament are exposed to chronic tensile stress, while lateral structures, including the iliotibial band, experience increased compressive forces. These abnormal mechanical stresses alter tissue length and tension, impair mechanoreceptor sensitivity (e.g., muscle spindles and Golgi tendon organs), and result in distorted sensory feedback to the central nervous system. Consequently, proprioceptive integration is compromised, leading to diminished postural control. This mechanism is supported by studies demonstrating that improvements in proprioception are directly associated with enhanced postural stability [37].

Furthermore, the elevated fear of falling observed in the knee valgus group extends beyond a simple behavioral response and reflects a state of internal instability. This phenomenon can be explained through the concept of motor self-efficacy [38] and the maladaptive cycle of “fear → reduced activity → muscular weakness → increased fall risk” [39]. Older adults who receive unreliable sensory and motor feedback from their lower limbs lose confidence in their movement capabilities, which promotes inefficient compensatory strategies such as excessive muscle co-contraction or reduced step length. These adaptations increase energy expenditure and accelerate fatigue, further exacerbating instability and fall risk [27].

Within this multifactorial framework, compensatory knee valgus in older adults should be considered not as a unidimensional structural deviation, but rather as a marker of a multisystem disorder encompassing muscular imbalances and abductor weakness [17], impaired somatosensory feedback [36], altered postural control strategies [40], and reduced motor self-efficacy accompanied by increased fear of falling [38].

This multidimensional interpretation supports the use of integrated interventions such as dynamic neuromuscular stabilization (DNS) exercises [41] and sensorimotor retraining programs [42], which simultaneously target strength, balance, and sensory integration. Accordingly, the findings of the present study emphasize that fall-prevention strategies for older adults should extend beyond isolated muscle strengthening and adopt a comprehensive corrective approach incorporating biomechanical alignment correction (pelvis and knee), closed-chain proprioceptive training, and interventions aimed at enhancing motor confidence. Consistent with the findings of Sadeghi et al. [25], optimization of lower limb alignment and core stability appears to be a key component in improving balance performance and reducing fall-related risk factors in older adults.

## Conclusion

The findings of the present study suggest that older adults with dynamic knee valgus may exhibit poorer balance, proprioception, and greater fear of falling. These findings indicate a potential association between dynamic knee valgus and factors related to balance and fall risk. However, causality cannot be inferred due to the cross-sectional design of the study. Additionally, neuromuscular factors such as muscle strength and activation were not directly assessed and may play a role in these associations. Therefore, in older adults who demonstrate dynamic knee valgus during squat performance, comprehensive assessment of movement patterns may be considered alongside traditional evaluations of balance and proprioception.

## Limitations

Several limitations should be acknowledged. First, participants were recruited using convenience sampling and were limited to older men from Urmia, which restricts the generalizability of the findings to women and to older adults from other geographical regions. Second, the relatively small sample size (*n* = 60) may have limited the statistical power to detect subtle differences, particularly in sensorimotor-related variables. Third, neither the assessors nor the participants were blinded, which may have introduced measurement or performance bias. Despite these limitations, the findings provide a useful foundation for future studies employing more rigorous methodologies, such as randomized controlled trials with active control groups and long-term follow-up.

Visual observation of dynamic knee valgus has been previously used as a practical clinical screening method in both research and clinical settings; however, its validity is influenced by standardized procedures and acceptable inter-rater and intra-rater reliability [43]. In the present study, knee alignment was assessed by a trained examiner with experience in movement analysis under standardized conditions. Nevertheless, formal reliability analysis was not performed, which should be acknowledged as a limitation of the study.

## Recommendations for future research

Given the observed association between compensatory knee valgus patterns and systemic weakness of both proximal (particularly hip abductors) and distal (ankle dorsiflexors) musculature, future research is recommended to examine the effects of corrective exercise interventions based on NASM–CES principles. Such interventions may include gluteus medius activation exercises, ankle pronation control strategies, and closed-chain squat movement pattern retraining. These effects should be investigated using randomized controlled trial (RCT) designs to determine their efficacy in improving balance, knee proprioception, and reducing fear of falling in older adults with knee valgus patterns. Additionally, as the present study included only older men, future research should examine these relationships in older women to enhance the generalizability of the findings.

## Data Availability

The datasets generated and analyzed during the current study are available from the corresponding author on reasonable request.
